# Involvement of Phosphorylation of Adenosine 5′-Monophosphate-Activated Protein Kinase in PTTH-Stimulated Ecdysteroidogenesis in Prothoracic Glands of the Silkworm, *Bombyx mori*

**DOI:** 10.1371/journal.pone.0063102

**Published:** 2013-05-09

**Authors:** Shi-Hong Gu, Yun-Chin Hsieh, Shun-Chieh Young, Pei-Ling Lin

**Affiliations:** Department of Zoology, National Museum of Natural Science, Taichung, Taiwan, Republic of China; New Mexico State University, United States of America

## Abstract

In this study, we investigated inhibition of the phosphorylation of adenosine 5′-monophosphate-activated protein kinase (AMPK) by prothoracicotropic hormone (PTTH) in prothoracic glands of the silkworm, *Bombyx mori*. We found that treatment with PTTH *in vitro* inhibited AMPK phosphorylation in time- and dose-dependent manners, as seen on Western blots of glandular lysates probed with antibody directed against AMPKα phosphorylated at Thr172. Moreover, *in vitro* inhibition of AMPK phosphorylation by PTTH was also verified by *in vivo* experiments: injection of PTTH into day 7 last instar larvae greatly inhibited glandular AMPK phosphorylation. PTTH-inhibited AMPK phosphorylation appeared to be partially reversed by treatment with LY294002, indicating involvement of phosphatidylinositol 3-kinase (PI3K) signaling. A chemical activator of AMPK (5-aminoimidazole-4-carboxamide-1-β-d-ribofuranoside, AICAR) increased both basal and PTTH-inhibited AMPK phosphorylation. Treatment with AICAR also inhibited PTTH-stimulated ecdysteroidogenesis of prothoracic glands. The mechanism underlying inhibition of PTTH-stimulated ecdysteroidogenesis by AICAR was further investigated by determining the phosphorylation of eIF4E-binding protein (4E-BP) and p70 ribosomal protein S6 kinase (S6K), two known downstream signaling targets of the target of rapamycin complex 1 (TORC1). Upon treatment with AICAR, decreases in PTTH-stimulated phosphorylation of 4E-BP and S6K were detected. In addition, treatment with AICAR did not affect PTTH-stimulated extracellular signal-regulated kinase (ERK) phosphorylation, indicating that AMPK phosphorylation is not upstream signaling for ERK phosphorylation. Examination of gene expression levels of AMPKα, β, and γ by quantitative real-time PCR (qRT-PCR) showed that PTTH did not affect AMPK transcription. From these results, it is assumed that inhibition of AMPK phosphorylation, which lies upstream of PTTH-stimulated TOR signaling, may play a role in PTTH stimulation of ecdysteroidogenesis.

## Introduction

**Figure 1:**
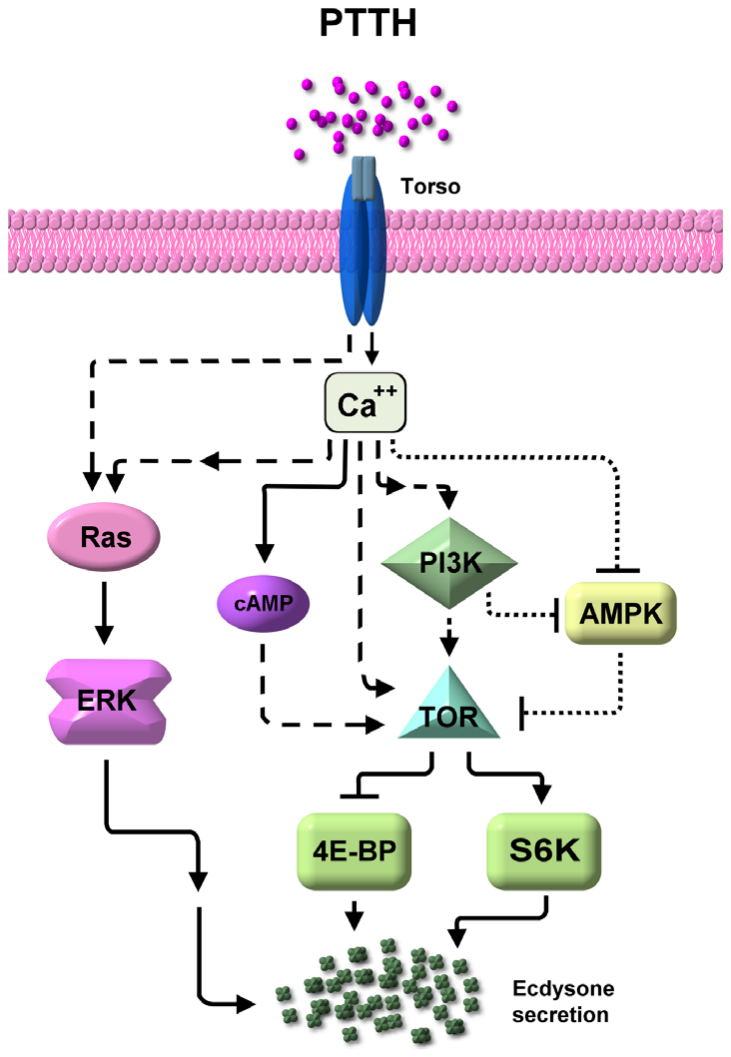
The signaling network involved in PTTH-stimulated ecdysteroidogenesis in prothoracic gland cells. Solid lines indicate demonstrated or highly likely relations; dashed lines indicate hypothetical interactions; dotted lines indicate the relationship demonstrated in the present study. PTTH, prothoracicotropic hormone; Ras, Ras GTP-binding protein; ERK, extracellular signal-regulated kinase; cAMP, cyclic adenosine monophosphate; PI3K, phosphatidylinositol 3-kinase; TOR, target of rapamycin; 4E-BP, eIF4E-binding protein; S6K, p70 ribosomal protein S6 kinase; AMPK, adenosine 5′-monophosphate-activated protein kinase (ref. 2, 3 and this work).

Ecdysteroids are the insect steroid hormones that are synthesized and secreted by the prothoracic glands upon stimulation by the brain neurohormone, prothoracicotropic hormone (PTTH) [1]–[3]. PTTH was first purified and sequenced from the silkworm, *Bombyx mori* [4]. Due to structure similarities, it was proposed that PTTH resembles mammalian growth factors [5]. Recently, it was demonstrated that Torso, a receptor tyrosine kinase that regulates embryonic terminal cell fate in *Drosophila melanogaster*, is a PTTH receptor [6]. PTTH appears to bind to its receptor to activate a signaling transduction network that up-regulates ecdysteroid synthesis [2], [3], [6] (Fig. 1). Cellular actions of PTTH are best understood for 2 lepidopteran insects, *B. mori* and *Manduca sexta*. Initial studies showed that cAMP and Ca^2+^ are intracellular second messengers involved in PTTH-stimulated ecdysteroidogenesis in both *M. sexta* [7]–[9] and *B. mori* [10], [11]. Later, evidence indicated that the phosphorylation of p70S6 kinase (S6K) and ribosomal protein S6 are activated upon PTTH stimulation in *M. sexta* [12]–[14]. Moreover, extracellular signal-regulated kinase (ERK) phosphorylation was demonstrated to be involved in PTTH’s stimulation of ecdysteroidogenesis [15]–[17]. It was recently further found that receptor tyrosine kinase is related to PTTH-stimulated ERK phosphorylation in *B. mori* prothoracic glands [17]. In addition, several other signaling pathways, such as tyrosine kinase, protein kinase C, the proteins involved in endosomal trafficking and constituents of the cytoskeleton, as well as the phosphorylation and translation of Halloween protein spook, appear to be involved in PTTH-stimulated ecdysteroidogenesis [18]–[20]. Finally, our recent study showed that the signaling of phosphatidylinositol 3-kinase (PI3K)/the target of rapamycin (TOR) is involved in the stimulation of *B. mori* prothoracic glands by PTTH [21], [22].

In eukaryotic cells, adenosine 5′-monophosphate-activated protein kinase (AMPK) is a key regulatory enzyme of cellular energy homeostasis and is involved in regulating a diverse range of metabolic pathways [23]–[26]. The AMPK protein complex consists of a catalytic α-subunit and regulatory β- and γ-subunits [23], [25]. AMPK activity is regulated allosterically by AMP and through phosphorylation at Thr172 in the activation loop of the α-subunit. AMPK regulates carbohydrate, lipid, and protein metabolism via its effects on multiple signaling pathways and thereby suppresses ATP-demanding processes and activates ATP-repleting pathways [23]–[26]. AMPK is activated during states of energy stress such as hypoxia, glucose starvation, and physical exercise. Protein synthesis, a major consumer of ATP in mammalian cells, is inhibited upon AMPK activation. Recent studies showed that one of the major downstream signaling pathways regulated by AMPK is the TOR signaling pathway [26]. The acute metabolic consequences of AMPK activation on protein homeostasis are mediated in part by the tuberous sclerosis complex (TSC)1 gene product and TSC2, which act upstream of the TOR pathways. Increased TSC2 phosphorylation and enhanced formation of the TSC1/TSC2 heterodimer both negatively regulate TOR activity [27]. In *Drosophila*, single genes encoding homologues of the α, β, and γ subunits of mammalian AMPK were identified [28]. *Drosophila* AMPK is highly similar to mammalian AMPK, as it is formed via a heterotrimeric complex, is activated by AMP, and has many of the same targets, including acetyl-CoA carboxylase (ACC) [28]. Recently, it was demonstrated that reduced AMPK signaling in *Drosophila* through RNAi knockdown leads to hypersensitivity to starvation conditions as measured by lifespan and locomotor activity [29].

In the present study, we investigated the involvement of AMPK signaling in PTTH-stimulated ecdysteroidogenesis in *B. mori* prothoracic glands. Our results showed that PTTH inhibited the phosphorylation of AMPK both *in vitro* and *in vivo*. Moreover, PI3K appeared to be partially involved in PTTH-inhibited AMPK phosphorylation. The AMPK activator, 5-aminoimidazole-4-carboxamide-1-β-D-ribofuranoside (AICAR), prevented PTTH-inhibited AMPK phosphorylation and greatly inhibited PTTH-stimulated ecdysteroidogenesis, suggesting that AMPK signaling is indeed involved in PTTH-stimulated ecdysteroidogenesis of prothoracic glands in *B. mori*.

## Materials and Methods

### Experimental Animals

Larvae of the F1 racial hybrid, Guofu × Nongfong, were reared on fresh mulberry leaves at 25°C under a 12-h light: 12-h dark photoperiod. Newly-ecdysed last instar larvae were collected and used for each experiment.

### Reagents and Antibodies

Recombinant *B. mori* PTTH (PTTH) was produced by infection of *Spodoptera frugiperda-*SF21 cells with the vWTPTTHM baculovirus as described previously [30]. The same stock solution of PTTH as that previously reported [16], [30], [31] was used in the present study. In the present study, the extracellular fluid from cells infected with vWTPTTHM was used as the PTTH source, and it was diluted 500 times with medium except for the experiments on the dose-dependent effects of PTTH and with *in vivo* injections. Each incubation (50 µl) contained about 0.15 ng PTTH. Grace’s insect cell culture medium was purchased from Invitrogen (Carlsbad, CA, USA). The mitogen-activated protein kinase (MAPK)/extracellular signal-regulated kinase (ERK) kinase (MEK) inhibitor (U0126), a PI3K inhibitor (LY294002), a cell-permeable AMPK activator (AICAR), and an AMPK inhibitor (compound C) were purchased from Calbiochem (San Diego, CA, USA).

Anti-phospho-AMPKα (Thr172), anti-phospho-4E-BP1 (Thr37/46), anti-phospho-ERK, anti-ERK and anti-α-tubulin antibodies were purchased from Cell Signaling Technology (Beverly, MA, USA). The anti-phospho-p70 S6 kinase (Thr412) antibody was purchased from Upstate (Lake Placid, NY, USA). A horseradish peroxidase (HRP)-linked goat anti-rabbit second antibody was purchased from PerkinElmer Life Sciences (Boston, MA, USA).

### *In Vitro* Incubation of Prothoracic Glands, Radioimmunoassay (RIA) of Ecdysteroids, and *In Vivo* Injection of PTTH

Considering the sensitive response to PTTH in prothoracic glands from day 7 last instar larvae, glands from this stage were dissected in lepidopteran saline [16]. Following dissection, the saline was replaced with fresh medium (± any inhibitors or AICAR), and a 30-min preincubation period was initiated. After preincubation, glands were rapidly transferred to fresh medium (± experimental materials, such as an inhibitor or PTTH) and then incubated with gentle shaking. Incubation lasted 1 h. After incubation, glands were flash-frozen at −70°C for a subsequent sodium dodecylsulfate polyacrylamide gel electrophoresis (SDS-PAGE) analysis. Ecdysteroids released into the medium were determined by an RIA according to procedures described in a previous study [32]. The assay was calibrated with 20-hydroxyecdysone as the standard. The antiserum has an approximate binding ratio of 3∶1 for 20-hydroxyecdysone to ecdysone [32]. The detection limit of the RIA was 0.03 ng. To study the *in vivo* effect of PTTH on AMPK phosphorylation, day 7 last instar larvae were injected with 10 µl saline containing 1 µl of the original PTTH solution. Larvae injected with 10 µl saline were used as the controls.

### Western Blot Analysis

**Figure 2:**
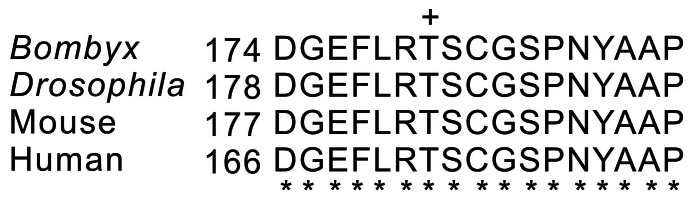
Comparison of predicted N-terminal amino acid sequences of the phosphorylation domains of *Bombyx* AMPK with their counterparts from *Drosophila*, mouse, and human. The plus sign (+) indicates a conserved amino acid residue (Thr) that is phosphorylated. Asterisks (*) indicate identical amino acids. AMPKs of various species were cited from the NCBI, and amino acid sequences were registered with the following accession numbers: ABQ62953.1 (*Bombyx*), AF020310.1 (*Drosophila*), AAB95475.1 (mouse), and AAB32732.1 (human).

Phospho-specific antibodies were developed and are commercially available for human AMPKα phosphorylated at Thr172 (cat. no. 2535, Cell Signaling Technology), as phosphorylation is known to be essential for the activity of these enzymes. Comparison of deduced amino acid sequences of the phosphorylation domain of *Bombyx* AMPKα with their counterparts from *Drosophila*, human, and mouse showed identical phosphorylation sites, indicating high conservation among species (Fig. 2). Conservation of phosphorylation sites among species suggests the possibility that commercial antibody against mammalian kinase can successfully be used to investigate *Bombyx* AMPK phosphorylation. Therefore, a commercial polyclonal antibody against mammalian AMPKα phosphorylated at Thr172 was used in the present study. In addition, because of the presence of conserved phosphorylation sites among species in ERK and TOR signaling, the same anti-phospho-ERK (Thr202/Tyr204), anti-phospho-4E-BP1 (Thr37/46), anti-phospho-p70 S6 kinase (Thr412), and anti-ERK antibodies as those previously reported [16], [17], [21], [22] were used to detect ERK and TOR signaling. The signals obtained with the anti-α-tubulin antibody were used as an internal reference. The Western blot analysis for each protein’s phosphorylation followed protocols as described in previous studies [16], [17], [21], [22], [33].

### Treatment with Lambda Protein Phosphatase

To ensure that the immunoreactive signals were specific for the phospho-antibody, gland lysates were treated with lambda protein phosphatase (New England Biolabs, Beverly, MA, USA), which removes phosphates from serine, threonine, and tyrosine residues [34]. Glands were directly lysed in phosphate buffer (50 mM Tris-HCl (pH 7.5), 100 mM NaCl, 2 mM DTT, 0.1 mM EGTA, and 0.01% Brij 35) with an added protease inhibitor cocktail (Sigma, St. Louis, MO, USA) according to the manufacturer’s instructions. Gland lysates were incubated at 30°C for 30 min with or without 1000 units of lambda protein phosphatase in 50 µl of reaction buffer. At the end of the incubation period, the sample was boiled for 5 min in an equal volume of SDS sample buffer and stored at −70°C until the SDS-PAGE analysis.

### RNA Extraction and Quantitative Real-time Polymerase Chain Reaction (qRT-PCR)

Three batches of total RNAs were prepared independently from 4∼6 *Bombyx* prothoracic glands for each time point. Total RNAs from prothoracic glands were extracted using the TRI Reagent (Molecular Research Center, OH, USA) and contaminating genomic DNA was removed by DNase I treatment according to the manufacturer’s protocol. The quantity of extracted RNA was assessed with a UV1101 photometer (Biotech, Cambridge, UK), and the ratio of 260/280 was 1.8∼2.0. First-strand complementary DNA (cDNA) was synthesized using an iScript cDNA synthesis kit (BIO-RAD, CA, USA).

A qRT-PCR was carried out in a 20-µl reaction volume containing 10 µl of SYBR1 Green Realtime PCR Master Mix (BIO-RAD), 2 µl of a first-strand cDNA template, and 8 µl of the primers. The iQ5 Real-Time PCR Detection System (Bio-Rad) was used according to the manufacturer’s instruction, and melting curve analysis was applied to all PCR reactions to ensure homogeneity of the amplified product. Only a single melting peak was found for all transcripts. RT-PCR primers were designed according to parameters (no primer dimers and a product length of no more than 200 bp) outlined in the manual of the SYBR1 Green Realtime PCR Master Mix. The annealing temperature for all reactions was 59.5°C. Transcript levels were normalized to the *Bombyx* ribosomal protein 49 (rp49) mRNA levels. The qRT-PCR reactions were performed using the following primers: AMPKα forward, 5′-CTGGAAGTGTTCCGAGCCAT-3′, AMPKα reverse, 5′-TACTCTGACGTGGTACGGGT-3′, AMPKβ forward, 5′-AAAGATGCTAACAAATCG-3′, AMPKβ reverse, 5′-GTTCAGTGTATTCAATATCA-3′, AMPKγ forward, 5′-GATGTACTACACGAGCCCCG-3′, AMPKγ reverse, 5′-TAGACGATGGATGCGGTTCG-3′, and RP49 forward, 5′-CAGGCGGTTCAAGGGTCAATAC-3′, RP49 reverse, 5′-TGCTGGGCTCTTTCCACGA-3′.

## Results

### Expression of AMPK

**Figure 3:**
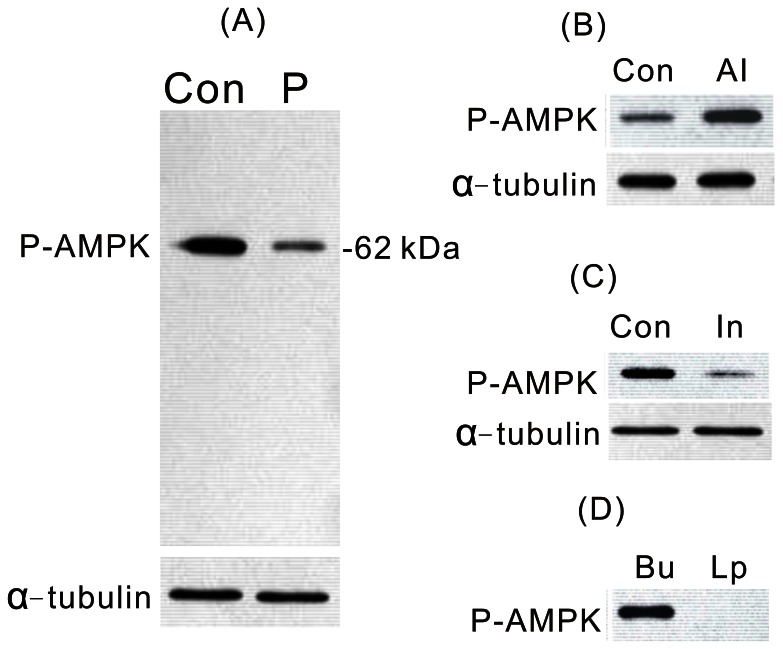
Effects of PTTH (A), AICAR (B), compound C (C), and lambda protein phosphatase (D) on AMPK phosphorylation. Con, glands incubated in control medium; P, glands incubated in medium containing PTTH; Al, glands incubated in medium containing 1 mM AICAR; In, glands incubated in medium containing 10 µM compound C; Bu, glands treated with buffer only; Lp, glands treated with lambda protein phosphatase. Results shown are representative of two independent experiments.

As the first step in studying the involvement of AMPK in PTTH signal transduction in silkworm prothoracic glands, AMPK phosphorylation of *B. mori* prothoracic glands was analyzed by immunoblotting with anti-phospho-AMPKα antibody, which only recognizes the phosphorylated form. Fig. 3A shows whole blots of lysates of prothoracic glands. A strongly immunoreactive protein with a molecular weight (MW) of about 62,000 was readily detectable in the lysate of a prothoracic gland from a day 7 last instar larva. The recombinant PTTH greatly inhibited AMPK level phosphorylated at Thr172. Phosphorylation of this site was shown to be necessary for activation of AMPK [35]. When gland lysates were incubated with lambda protein phosphatase prior to electrophoresis, the immunoreactivity detected by the antibody directed against mammalian anti-phospho-AMPKα (Thr172) was virtually eliminated (Fig. 3D), indicating that the above antibody, raised against mammalian sequences, indeed recognized a phosphorylated epitope in *B. mori* gland lysates. Further evidences that the phosphorylated protein is AMPK and that the antibody specifically recognizes AMPK, comes from use of the AMPK inhibitor, compound C, and the AMPK activator, AICAR. As shown in Fig. 3C, when prothoracic glands from day 7 last instar larvae were treated with compound C, *in vitro*, a great decrease in antigen recognition by the anti-phospho-AMPKα antibody was found. In contrast, treatment with AICAR greatly increased the immunoreactivity (Fig. 3B).

### PTTH Inhibited AMPK Phosphorylation in Time- and Dose-dependent Manners

**Figure 4:**
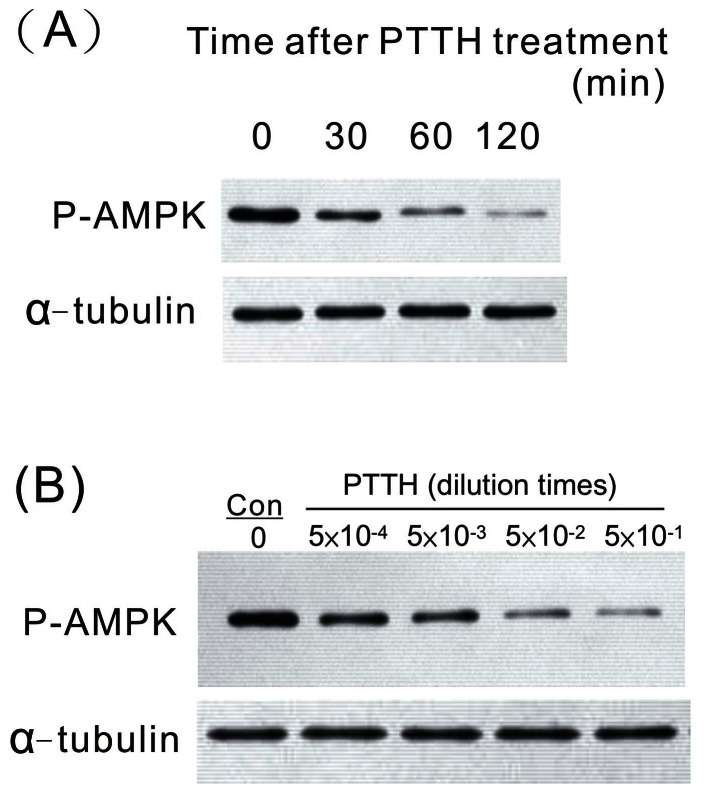
Time- and dose-dependent inhibitory effects of AMPK phosphorylation by PTTH. Prothoracic glands were either treated with PTTH for the indicated time points (A), treated with indicated concentrations of PTTH, or incubated with control medium (con) for 60 min (B). Results shown are representative of three independent experiments.

The effect of PTTH on AMPK phosphorylation was studied in greater detail by examining the level of AMPK phosphorylated at Thr172. Inhibition of AMPK phosphorylation by PTTH occurred after 30 min, and the inhibition persisted for 120 min (Fig. 4A). Fig. 4B shows the dose-dependent inhibitory effect of PTTH on AMPK phosphorylation. The total protein level was also checked using an anti-α-tubulin antibody, and results showed that it did not change with stimulation by PTTH. In addition, no inhibition of AMPK phosphorylation was observed when prothoracic glands were treated with extracellular fluid from cells infected with wt AcMNPV [30], indicating the specificity of the recombinant PTTH (data not shown).

### *In Vivo* Inhibition of AMPK Phosphorylation of Prothoracic Glands by PTTH

**Figure 5:**
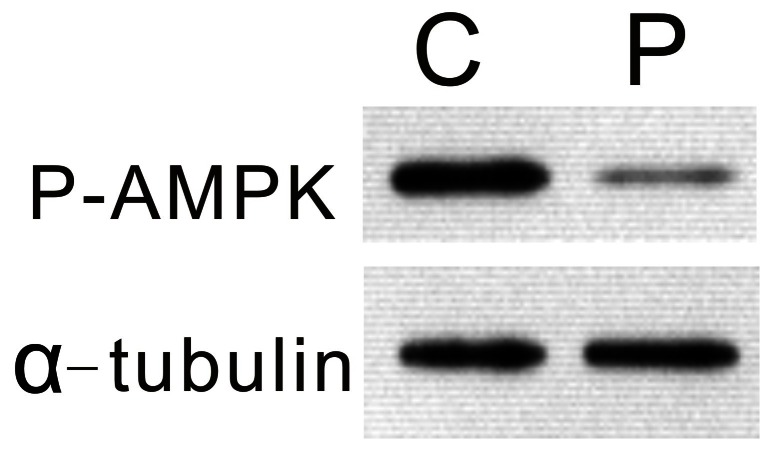
*In vivo* inhibition of AMPK phosphorylation of prothoracic glands by PTTH. P, larvae injected with saline containing PTTH; C, larvae injected with saline only. Results shown are representative of three independent experiments.

The above results clearly showed the *in vitro* inhibition of AMPK phosphorylation of silkworm prothoracic glands by PTTH. We further examined the *in vivo* effect of AMPK phosphorylation of the prothoracic glands by PTTH. Larvae from day 7 last instars were injected with PTTH. Thirty minutes later, the prothoracic glands were quickly dissected out, and AMPK phosphorylation was examined and compared to that of control larvae. Fig. 5 shows that a PTTH injection greatly decreased AMPK phosphorylation compared to that of the controls, verifying the *in vitro* inhibition of AMPK phosphorylation.

### PTTH-inhibited AMPK Phosphorylation is Mediated through a PI3K-dependent Pathway and is Independent of ERK Signaling

**Figure 6:**
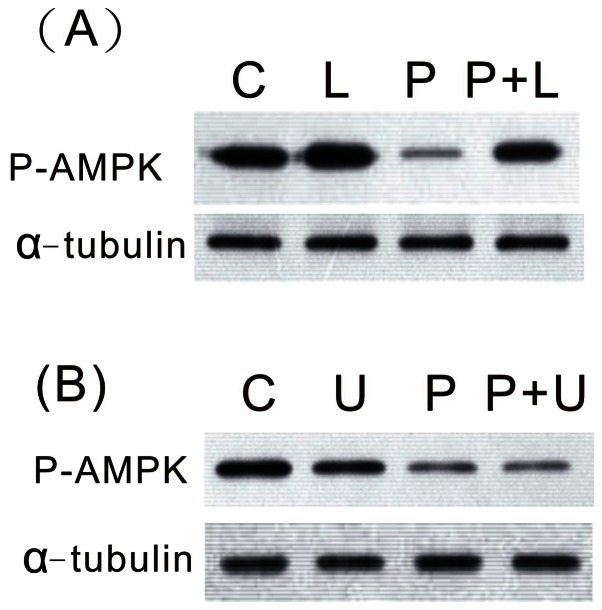
Effects of LY294002 (A) and U0126 (B) onPTTH-inhibited AMPK phosphorylation. C, glands incubated in control medium; P, glands incubated in medium containing PTTH only; L, glands incubated in medium containing 50 µM LY294002 only; P+L, glands incubated in medium containing both PTTH and LY294002; U, glands incubated in medium containing 10 µM U0126 only; P+U, glands incubated in medium containing both PTTH and U0126. Results shown are representative of three independent experiments.

Our previous study showed that treatment with the PI3K inhibitor, LY294002, greatly inhibited PTTH-stimulated ecdysteroidogenesis in *B. mori*, indicating the involvement of PI3K in ecdysteroidogenesis [21]. In addition, activation of ERK phosphorylation by PTTH was also confirmed in both *M. sexta* [15] and *B. mori* [16]. We further examined whether PTTH-mediated inhibition of AMPK phosphorylation occurs through the PI3K or ERK pathway. To test this, isolated prothoracic glands were pre-treated with either LY294002, or the specific MEK inhibitor, U0126, followed by treatment with PTTH for 60 min. Fig. 6 shows that preventing ERK activation by U0126 elicited no effect on PTTH-mediated inhibition of AMPK phosphorylation at Thr172, whereas LY294002 partially inhibited this response.

### Effects of AICAR Treatment on Basal and PTTH-regulated AMPK Phosphorylation and Ecdysteroidogenesis

**Figure 7:**
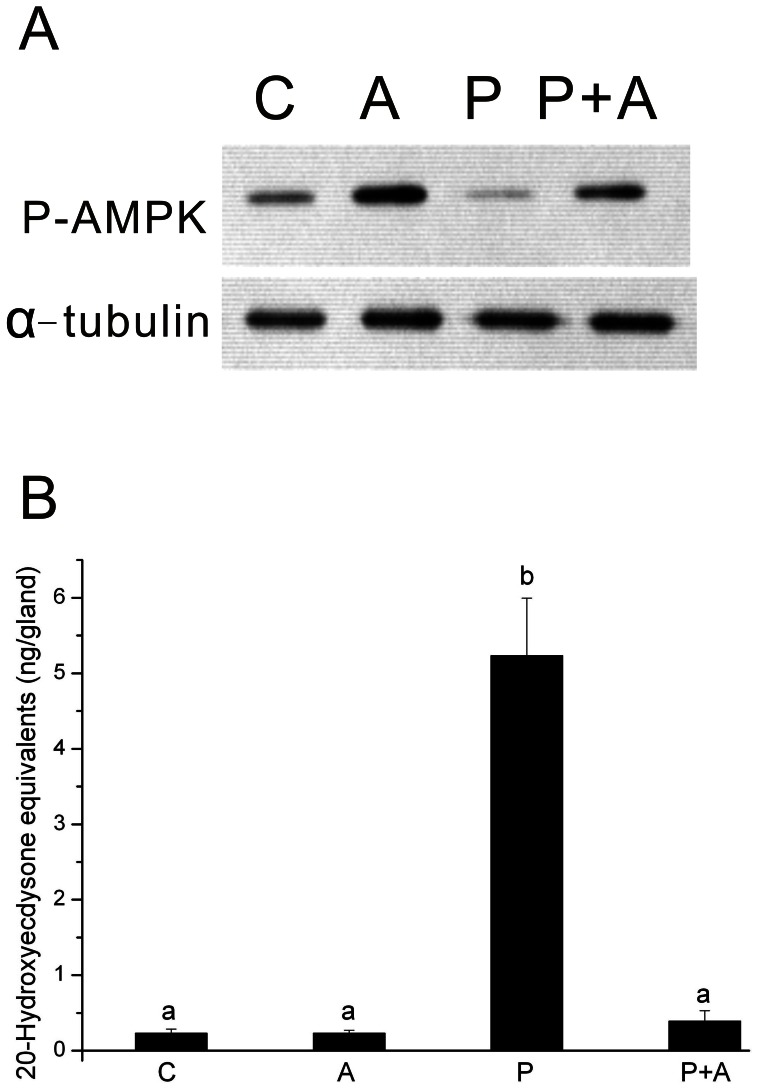
Effects of AICAR on PTTH-regulated AMPK phosphorylation (A) and ecdysteroidogenesis (B). C, glands incubated in control medium; P, glands incubated in medium containing PTTH only; A, glands incubated in medium containing 1 mM AICAR only; P+A, glands incubated in medium containing both PTTH and AICAR. Results shown for the Western blot analysis are representative of three independent experiments. Each bar represents the mean ± SEM (*N* = 8). Different letters above the bars indicate a significant difference (ANOVA followed by Tukey’s multiple-comparisons test, *p*<0.01).

The above results clearly showed that AMPK phosphorylation in prothoracic glands is inhibited by PTTH through the PI3K signaling pathway. To further examine whether this PTTH-mediated inhibition of AMPK phosphorylation is necessary for PTTH-stimulated ecdysteroidogenesis, we used AICAR, a pharmacological activator of AMPK [36]. To test this, isolated prothoracic glands were pre-treated with 1 mM AICAR, followed by treatment with PTTH for 60 min. Fig. 7A shows that treatment with 1 mM AICAR increased basal AMPK phosphorylation and reversed PTTH-mediated inhibition of AMPK phosphorylation, indicating its activation effect on AMPK. To examine whether treatment with AICAR inhibited PTTH-stimulated ecdysteroidogenesis, isolated prothoracic glands were pre-treated with 1 mM AICAR, followed by treatment with PTTH for 1 h. Ecdysteroids released into medium during the 1-h incubation were determined, and results (Fig. 7B) showed that treatment with 1 mM AICAR did not inhibit basal ecdysteroid secretion, but greatly inhibited PTTH-stimulated ecdysteroidogenesis. This result clearly shows that PTTH-mediated inhibition of AMPK phosphorylation is involved in ecdysteroidogenesis of prothoracic glands.

### Effects of AICAR Treatment on PTTH-stimulated TOR Signaling

**Figure 8:**
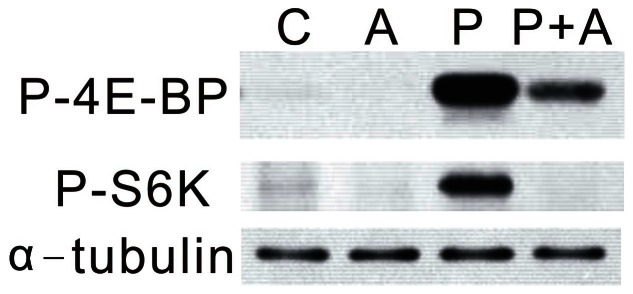
Effects of AICAR on PTTH-stimulated TOR signaling. C, Glands incubated in control medium; P, glands incubated in medium containing PTTH only; A, glands incubated in medium containing 1 mM AICAR only; P+A, glands incubated in medium containing both PTTH and AICAR. Results shown are representative of three independent experiments.

Our previous study showed that PTTH stimulates the phosphorylation of 4E-BP and S6K, two known downstream signaling targets of TORC1. In order to investigate whether AMPK is involved in PTTH/TOR signaling, the effects of AICAR on PTTH-stimulated phosphorylation of 4E-BP and S6K were examined. Prothoracic glands were pre-treated with 1 mM AICAR, followed by treatment with PTTH for 60 min, then phosphorylation of 4E-BP and S6K was determined. Fig. 8 shows that treatment with 1 mM AICAR abolished PTTH-stimulated phosphorylation of 4E-BP and S6K. These results indicate that TOR signaling is related to the PTTH-mediated AMPK signaling pathway.

### Effects of AICAR Treatment on PTTH-stimulated ERK Signaling

**Figure 9:**
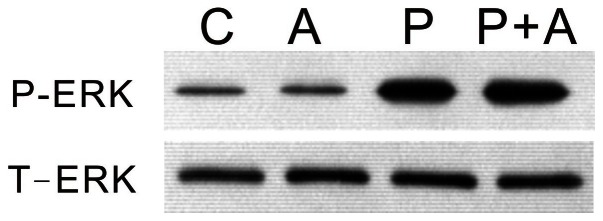
Effects of AICAR on PTTH-stimulated ERK phosphorylation. C, Glands incubated in control medium; P, glands incubated in medium containing PTTH only; A, glands incubated in medium containing 1 mM AICAR only; P+A, glands incubated in medium containing both PTTH and AICAR. Results shown are representative of three independent experiments.

In order to investigate whether AMPK is involved in PTTH/ERK signaling, the effect of AICAR on PTTH-stimulated ERK phosphorylation was examined. Prothoracic glands were pre-treated with 1 mM AICAR, followed by treatment with PTTH for 60 min; then ERK phosphorylation was determined. Fig. 9 shows that treatment with 1 mM AICAR did not affect PTTH-stimulated ERK phosphorylation. This result indicates that ERK is not a downstream signaling for PTTH-mediated AMPK pathway.

### Changes in Gene Expressions of AMPK upon PTTH Treatment

**Figure 10:**
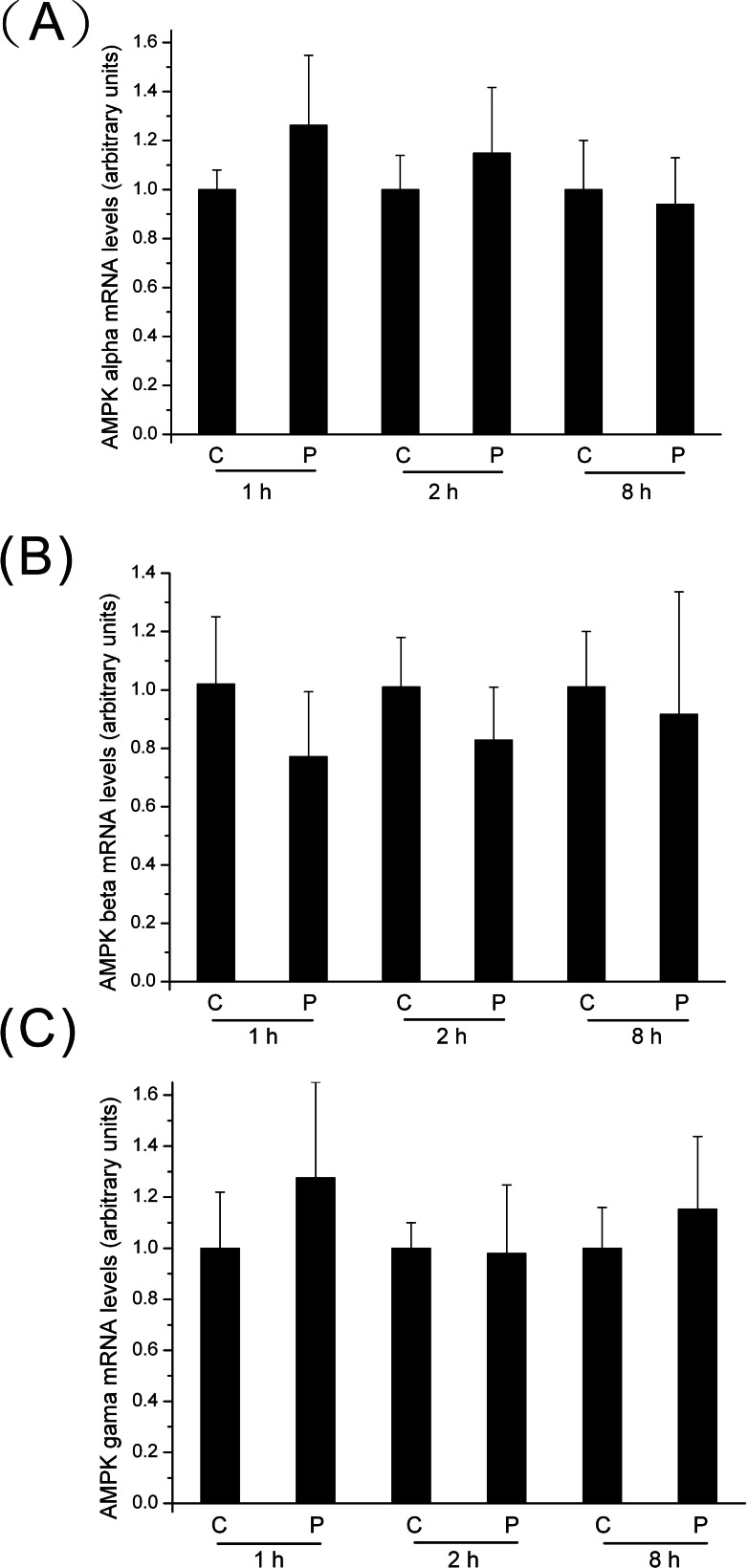
Changes in mRNA expression levels of AMPKα (A), β (B), and γ (C) upon treatment with PTTH. Prothoracic glands from day 7 last instar larvae were preincubated in medium for 30 min, then transferred to medium containing PTTH (P) or control medium (C). The incubation was maintained for 1, 2, and 8 h. After incubation, total RNA was extracted from the prothoracic glands, and mRNA expression levels of AMPK were determined by a qRT-PCR. Each bar represents the mean ± SEM of three separate assays.

A further experiment was conducted to examine changes in mRNA expression level of AMPKα, β, and γ upon PTTH treatment. As shown in Fig. 10, upon treatment with PTTH *in vitro*, no significant difference was detected in transcript levels of AMPKα, β, and γ between control glands and those treated with PTTH, when glands were incubated for either 1, 2 or 8 h, indicating that PTTH did not affect its transcript level.

## Discussion

The present study clearly shows that PTTH inhibited AMPK phosphorylation in time- and dose-dependent manners. Moreover, *in vitro* inhibition of AMPK phosphorylation of prothoracic glands by PTTH was also verified by *in vivo* experiments: injection of PTTH into day 7 last instar larvae greatly inhibited glandular AMPK phosphorylation. Pre-treatment with LY294002 partially prevented PTTH-inhibited phosphorylation of AMPK, indicating the involvement of PI3K signaling. The link between PTTH-inhibited AMPK phosphorylation and ecdysteroidogenesis was further demonstrated by AICAR, a chemical activator of AMPK. Pretreatment with AICAR increased both basal and PTTH-inhibited AMPK phosphorylation, and also greatly inhibited PTTH-stimulated ecdysteroidogenesis of prothoracic glands. These results clearly show that inhibition of AMPK phosphorylation plays a role in PTTH stimulation of ecdysteroidogenesis. Although the involvement of AMPK in regulating steroidogenesis was previously demonstrated in bovine granulose cells [37], to our knowledge, this is the first study to demonstrate the correlation between PTTH-stimulated ecdysteroidogenesis and the phosphorylation of AMPK in an insect system.

AMPK is a serine/threonine kinase and is highly conserved throughout eukaryotes [23]–[26], [38]. AMPK was first discovered as an activity that inhibited preparations of acetyl-CoA carboxylase (ACC) and 3-hydroxy-3-methylglutaryl-CoA reductase (HMG-CoA reductase, HMGR) and was induced by 5′-AMP [23]–[26]. It is thought of as a fuel gauge in the cell because it mediates a nutrient signaling pathway that senses the cellular energy status. AMPK activates a cascade of events within cells in response to the ever-changing energy charge of the cell. The role of AMPK in regulating the cellular energy charge places this enzyme at a central control point in maintaining energy homeostasis [23]–[26]. Recent evidence showed that AMPK regulates the energy balance at the whole body level via its modulation by hormones and cytokines [39]. In *D. melanogaster*, a homologue of AMP-activated protein kinase was identified and it was shown that it is sensitive to AMP and is activated by ATP depletion [28]. Deletion of the AMPK α-subunit in *D. melanogaster* was lethal, resulting in severe defects in embryonic cell polarity and the cell structure [40]. In addition, inhibition of *Drosophila* AMPK by RNAi in muscles reduced longevity and stress resistance [41]. In the present study, we identified that the phosphorylation of AMPK exists in *B. mori* prothoracic glands and that PTTH inhibits the phosphorylation of AMPK. In the mouse heart, it was reported that insulin treatment caused an increase in Akt phosphorylation and a decrease in AMPK phosphorylation [42]. A transgenic analysis showed that Akt activity negatively regulates AMPK phosphorylation [42]. Further evidence demonstrated that Akt is a key regulator of energy metabolism that inhibits AMPK [42]–[44]. Akt-deficient cells have reduced ATP levels and elevated AMPK activity, whereas cells expressing activated Akt have markedly elevated ATP levels and reduced AMPK activity [45]. Our previous study showed that PTTH stimulates PI3K signaling of prothoracic glands, leading to increased ecdysteroidogenesis [21]. The present finding that PTTH inhibits AMPK phosphorylation of prothoracic glands in a PI3K-dependent manner indicates that PI3K signaling is likely an upstream signal in the inhibition of AMPK phosphorylation.

In addition, our previous study showed that PTTH stimulates the phosphorylation of 4E-BP and S6K, two TOR target proteins and that PI3K/TOR signaling is involved in PTTH-stimulated ecdysteroidogenesis [22]. In the present study, it appears that activation of AMPK phosphorylation by treatment with AICAR inhibited PTTH-stimulated ecdysteroidogenesis of prothoracic glands. The inhibition of PTTH-activated TOR signaling by AICAR appears to be involved in inhibition of PTTH-stimulated ecdysteroidogenesis. This result implies that inhibition of AMPK phosphorylation by PTTH is related to PTTH-stimulated TOR signaling pathway. In mammalian systems, it is well documented that AMPK activation reduces protein synthesis by down-regulating TOR signaling [46]. It is assumed that the PI3K/AMPK/TOR signaling pathway may be highly conserved between insects and vertebrates. To our knowledge, this is the first study to demonstrate that AMPK signaling is involved in ecdysteroidogenesis in an insect system.

PTTH was shown to increase ecdysteroidogenesis of prothoracic glands through an ERK signaling pathway [3], [15], [16]. Our recent study further showed that PI3K/TOR and ERK signaling are two distinct signaling pathways involved in PTTH-stimulated ecdysteroidogenesis in *B. mori* prothoracic glands [22]. In the present study, we found that U0126, which is known to block ERK activation [16], did not affect PTTH-mediated inhibition of AMPK phosphorylation (Fig. 5B). This result rules out the role of ERK signaling in PTTH-mediated AMPK regulation. In addition, treatment with AICAR did not affect PTTH-stimulated ERK phosphorylation (Fig. 8), indicating that ERK signaling is not a downstream signal of the PTTH-mediated AMPK pathway. In addition, our present study also showed that PTTH did not exert its action at the transcriptional level of AMPKα, β, and γ, implying that the AMPK mRNA level is not related to PTTH-inhibited AMPK phosphorylation.

Taken together, the present study shows that AMPK, which so far is known as a controller of energy balance in cells, can also be regulated by PTTH in *B. mori* prothoracic glands. Based on this study and our previous studies, it is reasonable to conclude that PTTH increases ecdysteroidogenesis in *B. mori* prothoracic glands through an ERK-dependent pathway, and by regulating the AMPK signaling pathway through a PI3K-dependent pathway. In addition, pre-treatment with AICAR only partially prevented PTTH-stimulated 4E-BP phosphorylation, indicating AMPK-independent TOR signaling pathways.
